# The Impact of Redox Mediators on the Electrogenic and Physiological Properties of *Synechocystis* sp. PCC 6803 in a Biophotovoltaic System

**DOI:** 10.1002/cssc.202402543

**Published:** 2025-04-25

**Authors:** Jianqi Yuan, Yu Bai, Claudius Lenz, Vincent Reilly‐Schott, Hans Schneider, Bin Lai, Jens Olaf Krömer

**Affiliations:** ^1^ Department of Microbial Biotechnology Helmholtz Centre for Environmental Research – UFZ Permoserstraße 15 04318 Leipzig Germany

**Keywords:** 1,4‐benzoquinone, DUAL‐KLAS‐NIR, extracellular electron transfer, ferricyanide, metal‐organic complex

## Abstract

Biophotovoltaics (BPV) is a novel biohybrid solution to utilize solar energy potentially at high energy efficiency, by exploiting the water splitting in oxygenic photoautotrophs and electrochemical electron harvest. Unlike model electrogens, known phototrophic microbes benefit from redox mediators for extracting the photosynthetic electrons and transferring them to the external electron sink for further utilization. In this work, three representative mediators, i.e., 1,4‐benzoquinone (BQ), [Co(bpy)_3_]^2+^ (CoBP), and ferricyanide, are chosen and systematically evaluated for their impacts on the microbial physiology and electrogenic activity of *Synechocystis* sp. PCC6803. This work aimed to generate a knowledge base to guide future mediator selection and design. The results suggest ferricyanide remains the best option, as being the only mediator that promoted long‐term current output. However, both BQ and CoBP produce higher current densities than ferricyanide, albeit only for a short time. Comprehensive analysis of the photosystem using fluorometric methods suggests that BQ strongly increases the PQ/PQH_2_ ratio, while CoBP inhibits the electron flow from plastoquinone to photosystem I at high concentrations. Both mediators interrupt the photosynthetic electron flow and consequently cell growth. Eliminating the contribution of storage carbon to the intracellular electron flux demonstrates that all three chemicals can extract electrons originating from water splitting.

## Graphical abstract

Redox mediators are essential to promote the electrogenic activity of cyanobacteria in biophotovoltaics. In this work, we systematically evaluates three representative mediators about their influence on the cyanobacterial growth, photosynthetic efficiency, and electrogenic activity. The results reveal distinct impacts of different mediators, despite of similar redox potentials, and provide knowledge basis for future mediator design and selection.

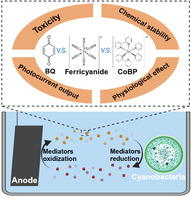

## 1 Introduction

Biophotovoltaics (BPV) is an emerging microbial electrochemical technology with unique benefits.^[^ ^1^ ^]^ It harnesses oxygenic photoautotrophic organisms, such as cyanobacteria and unicellular eukaryotic microalgae, to capture solar energy and convert it into renewable energy directly, i.e., electrical energy or hydrogen.^[^ ^2^ ^]^ Unlike conventional bioelectrochemical systems, BPV has been liberated from the dependence on organic substrates and uses light as its sole energy source by utilizing the highly efficient oxygenic photosystems.[^2^, ^3^] At the same time, a BPV can also fix CO_2_ during its operation,^[^ ^4^ ^]^ thus providing a low carbon footprint energy supply solution.

The BPV research is still in the early development stage. Compared to other concepts, e.g., producing green hydrogen from renewable energy using, e.g., photovoltaics (PV) and electrolyzers or photo‐(electro‐)chemical (PEC) water splitting techniques, BPV has a lower light‐to‐energy efficiency. Although the quantum efficiency of the photosystem can in theory reach 10–30%,^[^ ^5^ ^]^ providing a high upper boundary of the energy efficiency for the BPV concept, it typically only reaches less than 1% light‐to‐energy efficiency.^[^ ^1^, ^6^ ^]^ In contrast, commercial silicon‐based PV panels have reached well above 15% light harvesting efficiency, and for PEC light‐to‐energy efficiency is also above 10%.^[^ ^7^ ^]^ Typically, the photocurrent densities of PV and PEC are on the level of mA cm^−2^,^[7a]^ while BPV systems only reach levels in the range of μA cm^−2^ so far.^[2a]^ However, BPV has promising benefits in terms of energy and resource demands.^[^ ^8^ ^]^ The high light‐energy utilization efficiencies of PV and PEC rely on precious and rare metal‐based catalysts, many of which are even classified as EU Critical Raw Materials (e.g., gallium and tungsten). In contrast, BPV utilizes natural photosystems for water splitting, which are self‐regenerating using CO_2_ as carbon source. Another promising feature of the BPV concept is the thermodynamic energy gap that needs to be overcome. For instance, depending on how the photosynthetic electrons would be extracted, BPV needs to overcome an energy gap of up to 0.8 V to drive hydrogen evolution, which is at least one‐third less than the 1.2 V required for electrochemical water splitting.^[^ ^1^ ^]^ These unique features could potentially make BPV an interesting energy generation solution in the future, particularly considering the whole value chain and life cycle of the complete process.

Unlike model exoelectrogens such as *Shewanella* or *Geobacter*, the overwhelming majority of oxygenic photoautotrophic microorganisms lack multiheme cytochromes on the cell envelope, like OmcS and MtrCAB, which would facilitate direct electron transfer across the cell membrane to extracellular electron acceptors (i.e., the anode).^[2a]^ As a result, direct extracellular electron transfer (EET) is generally inapplicable or of extremely low efficiency in BPV systems.^[^ ^9^ ^]^ Although some studies reported very low direct EET in certain photoautotrophs,^[^ ^10^ ^]^ a critical uncertainty is the contribution of secreted redox metabolites to the observed current output.^[^ ^11^ ^]^ Consequently, in most cases, mediators were added in BPV systems to promote indirect EET.

**Figure 1:**
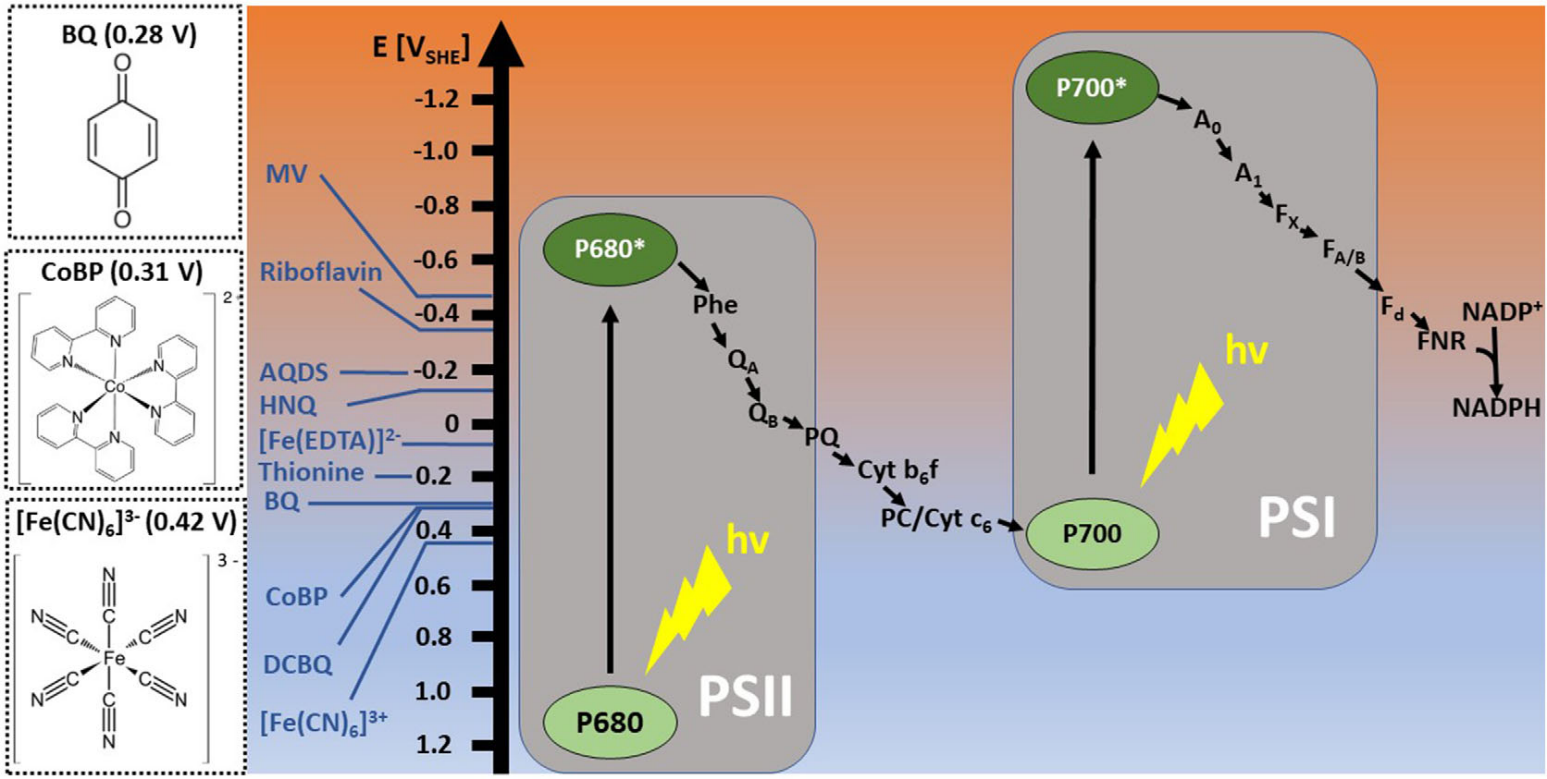
Z‐scheme of photosynthetic electron transfer chain, midpoint potential of various mediator molecules, and the chemical structures of three mediators studied in this work. P680* and P700* represent the excited states of P680 and P700, respectively. Phe denotes the pheophytin molecule; QA and QB represent quinone molecules, while PQ refers to the plastoquinone pool. Cyt b_6_f refers to the cytochrome b_6_f complex; PC and Cyt c_6_ correspond to the copper protein plastocyanin and cytochrome c_6_, respectively. A_0_ is a specialized chlorophyll a molecule, the primary electron acceptor of Photosystem I (PSI); A_1_ refers to phylloquinone. F_X_, F_A_, and F_B_ are three distinct iron‐sulfur centers. Fd stands for ferredoxin. MV, methyl viologen; AQDS, 9,10‐anthraquinone‐2,6‐disulfonate; HNQ, 2‐hydroxy‐1,4‐naphthoquinone; BQ, 1,4‐benzoquinone; DCBQ, 2,6‐dichloro‐1,4‐benzoquinone; [Fe(CN)_6_]^3‐^, ferricyanide; CoBP, [Co(bpy)_3_]^2+^; [Fe(EDTA)]^2‐^, ethylenediaminetetraacetatoferrate(^2‐^).

As illustrated in the Z‐scheme (**Figure** **1**), photosynthetic electrons, deriving from water splitting, move along the linear electron transfer chain, from low (negative) to high (positive) redox potentials. By utilizing various mediators with specific midpoint potentials and physicochemical properties, it would be possible to target particular points in the electron transfer chain.^[^ ^1^ ^]^ Generally, mediators with low midpoint potentials, e.g., methyl viologen or riboflavin, are thermodynamically less favored to extract electrons from the photosynthetic electron transportation chain, unless the photosystems could be directly targeted. Employing these mediators often results in low photocurrent generation efficiency.^[2a]^ Based on Marcus’ theory, a mediator with a higher midpoint potential could provide a stronger thermodynamic driving force to facilitate electron transfer and could in principle achieve a higher EET efficiency.

However, the physicochemical properties of redox mediators can be diverse, and physiological effects can be unpredictable.^[^ ^12^ ^]^ For example, 2,6‐dichloro‐1,4‐benzoquinone (2,6‐DCBQ) can penetrate *Chlamydomonas reinhardtii* and extract electrons from the photosynthetic electron transfer chain in the thylakoid membrane. It competes with the plastoquinone pool as the electron acceptor for photosystem II (PSII).^[^ ^13^ ^]^ When used as a mediator, 2,6‐DCBQ induces kinetic quenching in *Chlamydomonas reinhardtii*, resulting in a progressive photocurrent decline across different BPV setups.^[^ ^13^, ^14^ ^]^ Beyond quenching, many other quinones exhibit varying degrees of cytotoxicity due to their oxidizing properties.^[^ ^15^ ^]^ In contrast, ferricyanide cannot cross the (inner) membrane of cyanobacteria due to its hydrophilicity.[^8^, ^16^] Furthermore, methyl viologen was reported to be oxidized by oxygen, generating hydroxy superoxide that would cause strong oxidative stress to the microbes.[^8^, ^17^] Pyocyanin, a cell‐permeable phenazine, showed a fourfold increase in photocurrent generation compared to mediator‐free systems. However, the side reaction of oxygen reduction, due to the low midpoint potential of pyocyanin, competes with the photocurrent production.^[^ ^18^ ^]^ A thorough understanding of the effects of mediators on microbial physiology, as determined by their physicochemical properties, is essential for their effective application in BPV systems.

In this work, we selected three mediators with relatively high positive midpoint potentials to evaluate their electrochemical and physiological impacts on the cyanobacterium *Synechocystis* sp. PCC 6803 (hereafter *Synechocystis*) in a BPV system. The chosen compounds were 1,4‐benzoquinone (BQ), ferricyanide and [Co(bpy)_3_]^2+^ (CoBP). Their chemical structures are shown in Figure 1. BQ was selected as the representative of the quinone family, which is involved in electron transfer in living organisms and widely used as a mediator in bio‐electrochemical systems and enzymatic photo‐electrochemistry that employs photosystems.^[^ ^13^, ^19^ ^]^ This family includes variants such as 2,6‐dimethyl‐1,4‐benzoquinone (DMBQ) and 2,5‐dichlorobenzoquinone (2,5‐DCBQ). Notably, BQ exhibited superior photocurrent generation compared to the other five quinone derivatives when facilitating extracellular electron transfer (EET) using the purple bacterium *Rhodobacter capsulatus*.^[^ ^20^ ^]^ Ferricyanide is the most commonly used mediator in BPV systems^[^ ^21^ ^]^ and is used as a reference point. Finally, CoBP has a similar metal‐organic structure as ferricyanide but has a lower midpoint potential. It is a new mediator for BPV systems but has been validated for heterotrophs in bioelectrochemical systems.^[^ ^22^ ^]^

Various concentrations of each mediator were systematically tested in a BPV system. Growth inhibition on *Synechocystis* was used to evaluate cytotoxic effects, and optimal concentrations for maximizing bioelectricity generation while maintaining cell viability were identified. Additionally, a combination of fluorometric and spectroscopic techniques was employed to investigate the underlying mechanisms responsible for mediator‐induced growth inhibition. Furthermore, the intrinsic chemical stability of the mediators within the BPV system was evaluated to assess their long‐term viability for electron transfer. This study advances our understanding of the role of mediators and EET in BPV systems, providing a foundation for further optimization of mediator performance and system efficiency.

## 2 Results and Discussion

### 2.1 Effects of Mediators on the Growth and Electrogenic Activity of *Synechocystis*

A μM to mM range of concentrations was tested to evaluate the dual effects of mediators on the physiology and electrogenic activity of the *Synechocystis* in the BPV system.

**Figure 2:**
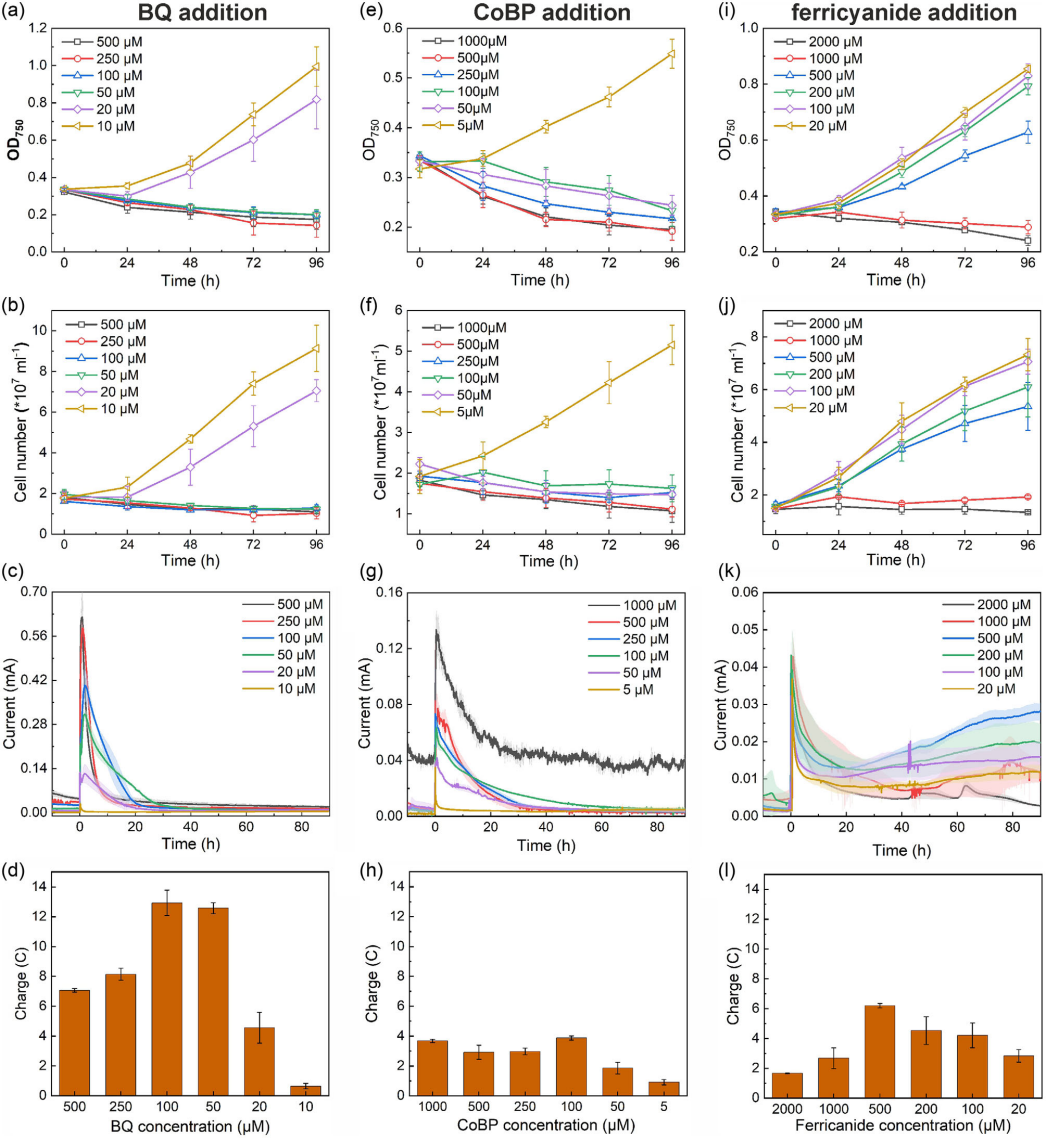
*Synechocystis* growth and bioelectric current output in a BPV system under varying concentrations of three redox mediators. a,e,i) The optical density OD_7__5__0_ of the cells over 4 days of cultivation in BPV. b,f,j) The corresponding cell numbers measured using Coulter Counter. c,g,k) The current profiles at different conditions, with the light‐shaded regions accompanying each current curve representing the standard deviations. d,h,l) The corresponding total charge output over the BPV batch, corresponding to the current profiles. Continuous illumination of 100 μmol photons m^−2^ s^−1^ was applied over the batch, and the reactors were inoculated at 0 h. Means and standard deviations are given (*n* = 3).

The addition of BQ inhibited cell growth but promoted a single peak current output, much higher compared to the other two mediators (**Figure** **2**a–d). The growth inhibition became more severe as the concentration increased (Figure 2a,b). No growth was observed at concentrations of 50 μM or above. In comparison, 2,6‐DCBQ, which has been shown to be nontoxic to *Synechocystis* at concentrations below 200 μM based on a cytotoxicity spot assay over 3 days,^[^ ^18^ ^]^ demonstrated a weaker inhibitory effect than BQ in this study. Moreover, 80% *Chlamydomonas reinhardtii* cells can still grow under 70 μM BQ,^[^ ^15^ ^]^ suggesting that *Synechocystis* is less resilient to BQ cytotoxicity. In contrast, the current output showed the opposite correlation with the concentration of the mediator (Figure 2c,d). The current output peaked shortly after inoculation, and the peak current increased as the BQ concentration increased, ranging from around 0.02 mA for 1 μM to over 0.62 mA at 500 μM. However, following the transient peak, the current output steadily declined to the background level and could not be recovered over 4 days of BPV cultivation. The lifespan of the current output at all tested concentrations was less than 30 h. This current decay phenomenon is similar to that commonly observed in 2,6‐DCBQ‐mediated systems,^[^ ^13^, ^14^, ^18^ ^]^ suggesting that the two chemicals share a similar underlying mechanism. Considering the overall charge output over the batch phases, the optimum concentration of BQ seems to be 50–100 μM, where the maximum electrons were extracted to the anode via the mediator (Figure 2d). Over 4 days of cultivation, the BPV system generated over 12 C electricity, despite the absence of growth.

The inhibition of growth was most severe for CoBP (Figure 2e,f). Here, already 5 μM of CoBP showed strong effects. The cell number increased from 2 × 10^7^ cells ml^−1^ to only about 5 × 10^7^ cells ml^−1^ in 4 days of cultivation. In the case of BQ, the final cell number reached about 9 × 10^7^ cells ml^−1^ even at a concentration of 10 μM. Similar to BQ, CoBP also enabled only a single peak current output but at a much lower value (Figure 2g,f). The current output reached its peak after inoculation and gradually dropped back to the baseline at around 30–40 h, similar to the observation with BQ. The charge output was correspondingly much lower, only reaching a maximum of 3.88 C with 100 μM of mediator.

In contrast to the above mediators, ferricyanide was much less toxic to cell growth (Figure 2i,j). The inhibitory effect also increased with increasing concentrations; however, the cell growth was only partially impaired even at a concentration of 500 μM. We only observed complete growth arrest at ferricyanide concentrations above 1 mM. In terms of electricity generation, ferricyanide mediated a similar current output to that of CoBP but lower than in the case of BQ. However, the current output was sustained much longer (Figure 2k,l). The peak current output after inoculation did not show a strong correlation to the ferricyanide concentrations, and despite slowly dropping, the current never reached the baseline level. After about 20 h of incubation, the current profiles for the systems with nonlethal concentrations of ferricyanide started to increase until the end of the batch. The observed current output over 80 h (Figure 2K) shows that photosynthetic activity of *Synechocystis* and interaction with the mediator has the potential to achieve long‐term current output, which could be further confirmed by Figure 6.

Generally, the addition of any mediator caused negative effects on the cell growth, even at low concentrations (see Table S1, Supporting Information), compared to the growth of *Synechocystis* without mediator.^[^ ^4^ ^]^ A long‐term current output unfortunately was only achievable with ferricyanide. Aiming at a balance between growth and long‐term current output, we consider ferricyanide to be the best of the three mediators. This aligns well with the fact that it is currently probably the most commonly used mediator in BPV research, and it is also widely used to promote the electrogenic activity of heterotrophs in bioelectrochemical system studies.[^22^, ^23^] In *Pseudomonas putida*, for instance, ferricyanide is most likely extracting electrons from the cytoplasmic membrane on the level of cytochrome c reductase.^[^ ^24^ ^]^ But such a reductase is not present in *Synechocystis*, and several publications have hypothesized that an unknown reductase(s) at the cell membrane could react with the ferricyanide for EET.[^8^, ^16^] Nevertheless, ferricyanide can at most penetrate the outer membrane and reach the periplasm;^[10b]^ thus, it is unlikely to directly interact with the main energy and redox machinery located in the intracellular thylakoid membranes (i.e., the photosystems and the photosynthetic electron transfer chain) in *Synechocystis*. This might be one of the reasons for the observed lower toxicity of this mediator, but also for the lower current output, since electrons have to travel to the cell envelope from thylakoid membrane for EET.

However, the performance of CoBP was unexpected. It shows almost identical performance as ferricyanide in influencing the microbial metabolism and in the EET mechanism while being introduced to *Pseudomonas putida*.[^22^, ^24^] But when applied in BPV, CoBP promotes, as described above, similar current levels, but the current profile lasts only for a short period and the cells are dying. Our CoBP was added as a perchlorate salt to the culture, raising the question, if this could also have an effect. The anion perchlorate is reported to have a strong inhibition effect on the photosynthesis of *Synechocystis* at concentrations of above 25 mM,^[^ ^25^ ^]^ which is hundreds of times higher than the lethal concentration of the mediator observed in this study. This suggests that the perchlorate is likely not the reason for impaired growth, but the cation CoBP ([Co(bpy)_3_]^2+^). The effects of this chemical on the photosystem are discussed further below.

The high current output for BQ follows our expectations based on literature. The quinone family (including BQ) has been demonstrated to be an excellent electron sink for photosystems in many in vitro studies,^[^ ^26^ ^]^ while the chemical structure of BQ also allows it to penetrate the cell membrane. However, the short‐term current output and interruption of cell growth at high concentration are not fully understood. At low concentration (i.e., 20 μM), the cells reached a comparable growth rate to that of ferricyanide. It was reported that BQ can act as a *Hill* reagent, changing the cell membrane permeability, and serving as the electron acceptor for the red algae *Porphyridum cruentum*.^[^ ^27^ ^]^ BQ may also play a similar role for *Synechocystis* in the BPV system, where the change of cell membrane structure and permeability might explain the high toxicity. However, even at low concentrations where the cells can still grow, the current output still only lasted for a short period, which cannot be explained by the toxicity effects of BQ.

### 2.2 Chemical Stability in BPV

While ferricyanide was stable under our BPV condition^[^ ^4^ ^]^ (Figure S1b, Supporting Information), the short‐term current output of BQ and CoBP questioned the stability of the chemicals in the BPV system where both light and anodic potential bias were present. CoBP is typically regarded as a highly stable redox chemical.^[^ ^28^ ^]^ We demonstrated previously that CoBP was stable in the chemically defined M9 medium,^[^ ^24^ ^]^ which is, from an electrochemistry perspective, comparable to the nBG11 medium used here. The total charge output from several oxidation–reduction cycles remained constant, demonstrating the total amount of redox chemical (i.e., CoBP) was the same over the batch. To further confirm its stability in nBG11 medium and under BPV conditions, we applied another electrochemical method, cyclic voltammetry (CV), to compare the voltammograms of the mediator before inoculation and after the end of the batch in BPV. The results indicated that there was no significant change in the CoBP concentration over 4 days in BPV (Figure S1a, Supporting Information), as the total peak currents did not differ significantly before and after the cultivation. Thus, the degradation of CoBP could not be the cause for the drop in the electrogenic activity of *Synechocystis*. It seems more plausible that a molecular mechanism somehow interrupts current output, even at low CoBP concentrations where cells are still able to grow. The underlying mechanism needs further investigation.

**Figure 3:**
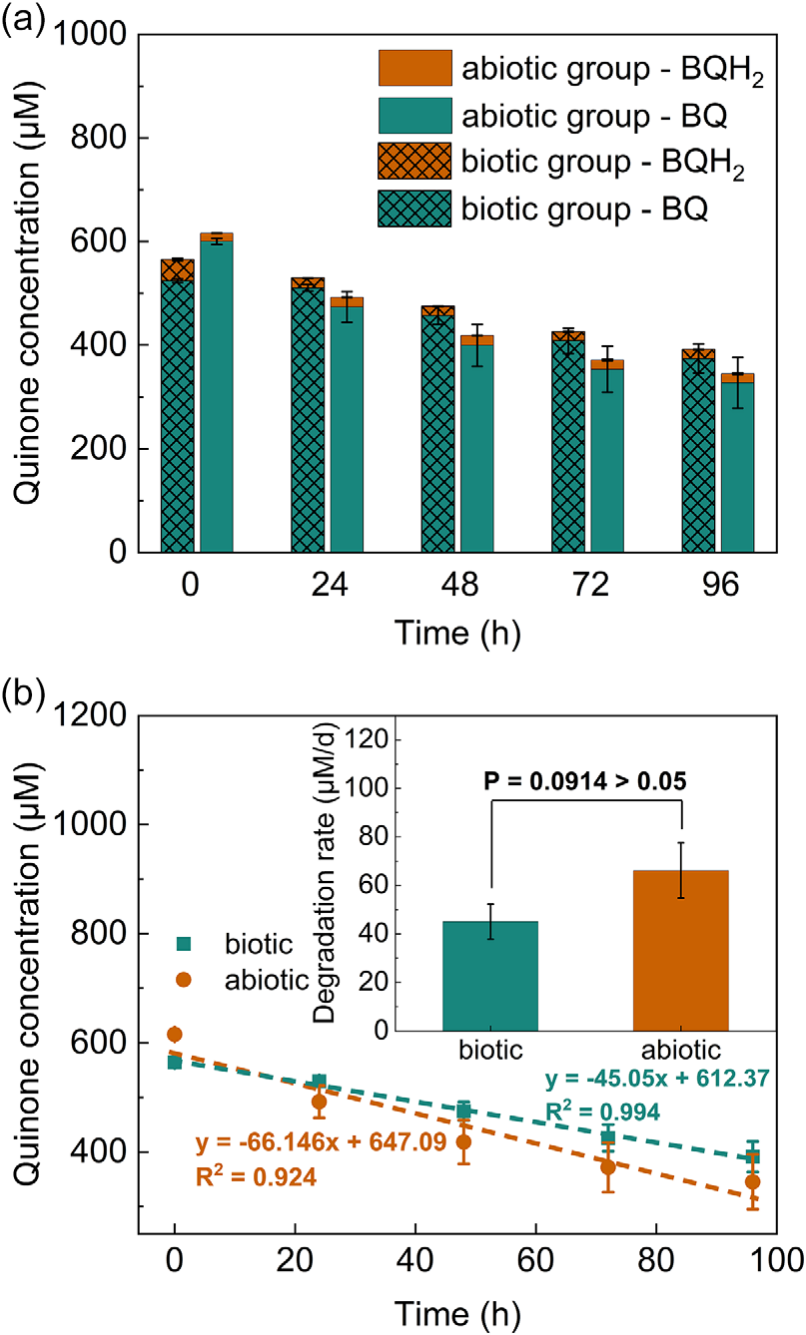
Concentration change of BQ in biotic and abiotic BPV reactors over 4 days of incubation. a) Concentration of BQ and its reduced form hydroquinone (BQH_2_). b) Fitted curves for total quinone concentrations (BQ & BQH_2_). The inset shows the fitted degradation rates and a comparison between biotic and abiotic systems. Means and standard deviations are presented (*n* = 3). *P* value was calculated based on analysis of variance (ANOVA) (*n* = 3).

BQ, however, was found to degrade over time under BPV conditions. The first parameter we tested was the influence of the biomass in the system. BQ can penetrate the cytosol because of its great lipophilicity.^[^ ^29^ ^]^ We added BQ to a BPV with *Synechocystis* (biotic group) and to an identical BPV setup without cells (abiotic group). The results are presented in **Figure** **3**. The concentration of BQ decreased in both scenarios, and the degradation rate under abiotic conditions was even higher. These results exclude the microorganism (or the interaction of BQ with the cells) as the reason for the degradation of BQ in our BPV system.

**Figure 4:**
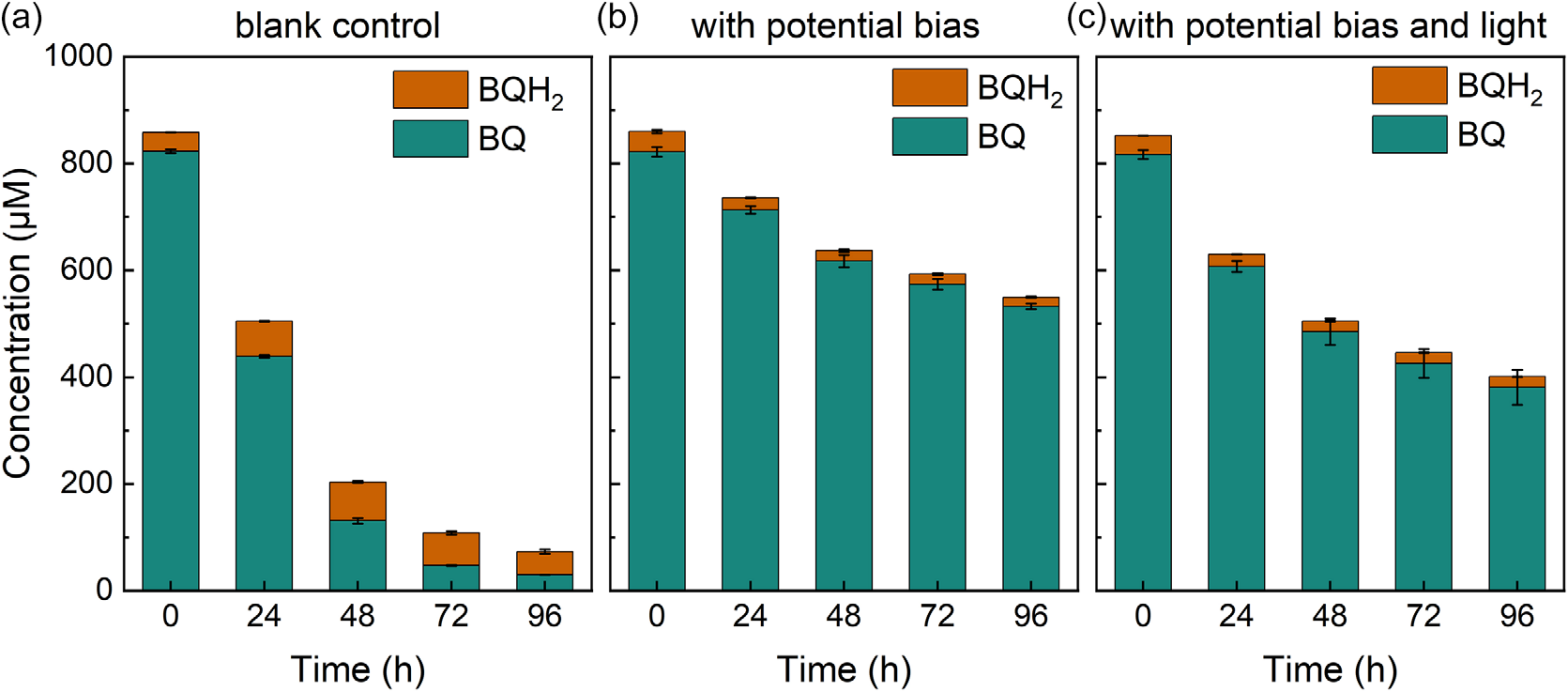
Effects of light and electrode potential on concentration of BQ in abiotic BPVs. Concentrations of oxidized BQ and reduced BQ (BQH_2_) over 4 consecutive days in BPV systems a) without potential bias under darkness; b) with 0.697 V potential bias under darkness; and c) with a 0.697 V potential bias under illumination. Means and standard deviations are presented (*n* = 3).

Next, we tested if the light or the applied electrical potential could affect the stability of BQ in BPV (**Figure** **4**). Abiotic BPV reactors were used, since, as shown above, *Synechocystis* did not affect the stability of the mediator. We evaluated three conditions: i) A BPV system with 0.697 V potential bias but without illumination, ii) a BPV system without illumination and without potential bias (blank control), and iii) a BPV system with 0.697 V potential bias and illumination with 100 μmol photons·m^−2^ s^−1^. By comparing the first two conditions, we found that the chemical concentrations decreased much faster in the blank control. Performing absorbance scans between 200 and 400 nm also showed that in the case of the blank control, absorbance spectra changed much more pronounced over time (Figure S9, Supporting Information). With potential bias on the anode, the chemical can be maintained mostly in the oxidized state (BQ); in the system without potential bias applied, the ratio between reduced and oxidized form of the redox couple was shifted to hydroquinone (BQH_2_) (Figure 4a), which could possibly be due to i) the electrochemical oxidation not occurring without potential bias and also ii) the auto‐oxidation of BQH_2_ to BQ by reacting with oxygen hardly happening at pH 8 or below.^[^ ^30^ ^]^ In the blank control, 858 μM of the mediator (including both BQ and BQH_2_ forms) declined to 73 μM after 4 days of operation, which suggests that the degradation can be mostly attributed to BQH_2_ degradation. Further comparative analysis of the first and third conditions suggests that illumination also facilitates the chemical degradation. With the presence of light, the chemical degradation is faster compared to the non‐illuminated BPV (Figure 4b,c), which could possibly be due to the photolysis of BQ. The white LED light used in this study generates a light spectrum in the visual range of 400–700 nm (see Figure S2, Supporting Information), and BQ was reported to have a weak absorption spectrum in the range of 400–600 nm.^[^ ^30^ ^]^ Although the photolysis of BQ is mainly facilitated by the UV light,^[^ ^31^ ^]^ the visual wavelength could also contribute to the slightly higher degradation rate of BQ while illuminated with light.

The chemical stability is likely a primary reason for the short‐term current output of BPV systems with BQ as the mediator. About 220 μM mediator, which is higher than most of the mediator concentrations tested in the BPV cultivations (Figure 2), is degraded over 24 h with the presence of potential bias and light (Figure 4c). This equals to about 9 μM h^−1^ if assuming a constant rate. However, it is also important to note that the current output drop starts at about 1–2 h after inoculation for all concentrations, which should be much earlier than the complete degradation of the chemicals, particularly at higher concentrations, given the averaged degradation rate above. This suggests a second effect coming from BQ on the current output profile, which remains to be investigated. The photoquenching effect was thought to be one possible reason, but is unlikely. Exogenous quinones like 2,6‐DCBQ, which are known to exhibit quenching effects, would lead to a fluorescence signal drop in the PAM measurement.^[^ ^13^, ^14^ ^]^ We did not observe this phenomenon in our PAM measurements (see Figure S4, Supporting Information). The fluorescence baseline stayed constant after the addition of BQ. Further investigation is needed to explore the specific effects of BQ on the photosystems.

### 2.3 Effect of BQ and CoBP on PQ Pool and PSI

The chemical stability analysis suggests both BQ and CoBP can cause unknown effects on the phenotype of *Synechocystis*, leading to the inability to export the photosynthetic electrons over longer times. To further investigate the potential mechanism, we applied PAM fluorometry and DUAL‐KLAS‐NIR to systematically evaluate if the redox state of the PQ pool and PSI were affected by the mediators.

**Figure 5:**
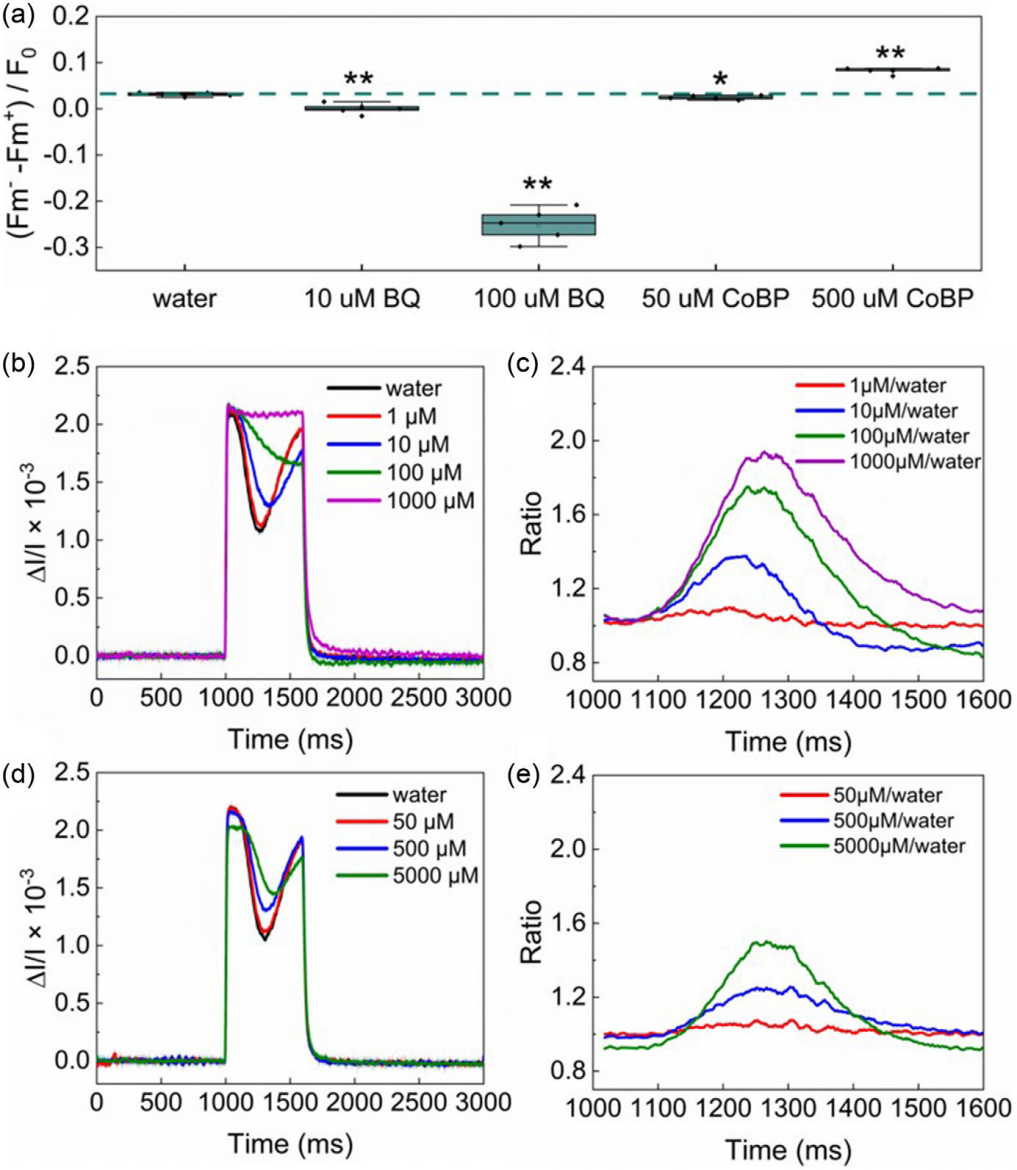
PQ pool and P700 redox status dynamic change of *Synechocystis* incubated at various concentrations of BQ and CoBP. a) Box plot of status state changes after the addition of two mediators at specific concentrations or the same volume of water (each case with five replicates). Black horizontal lines within the boxes represent the median, while small hollow squares indicate the mean. The green dashed line represents the median of the water‐added sample, used as a reference. Asterisks denote statistically significant differences compared to the “water” group, as determined by ANOVA (*P ≤ 0.05, **P ≤ 0.01). b) P700 behavior with different concentrations of BQ added. At 1000 ms, an actinic light pulse of 1350 μmol photons·m^−2^·s^−1^ was applied and lasted for 600 ms. A positive value means oxidation of P700, whereas negative value represents reduction. c) Normalized P700 redox change with different concentrations of BQ added during the 600 ms light pulse phase. d) P700 redox change with various concentrations of CoBP in the culture. e) Normalized P700 redox change with different concentrations of CoBP. All measurements in (b) and (d) were conducted 16 times repeatedly with 30 s darkness intervals, and the traces shown are the averages.

The redox state of the PQ pool, which is associated with the state transition of photosystems, can be assessed using PAM as described previously.^[^ ^32^ ^]^ Further details can also be found in Figure S4, Supporting Information. An increased fluorescence after mediator addition (Fm^+^) compared to the initial condition (Fm^−^) indicates that the PQ pool becomes more oxidized. At low concentrations, both BQ and CoBP (10 μM and 50 μM, respectively) lead to a more oxidized PQ pool (**Figure** **5**a), suggesting the photosynthetic machinery is more in a state, when the phycobilisomes are attached to PSII. Increasing the BQ concentration further enhances the oxidization effect (i.e., PQ/PQH_2_ ratio further increases), while a higher concentration of CoBP, in contrast, decreases the PQ/PQH_2_ ratio. Such a different trend indicates a distinct mechanism of BQ and CoBP on the photosynthetic electron flow, despite both of them extracting electrons into the extracellular space.

We further investigated the redox status of PSI using DUAL‐KLAS‐NIR, to better understand the effects of these two mediators on the photosystems. We monitored the dynamic changes of the redox state in the PSI reaction center (P700) at different mediator concentrations (Figure 5b–e). Upon activation of actinic light at 1000 ms, P700 is fully oxidized instantaneously and then is gradually reduced as it receives electrons from PSII (Phase 1). After about 300 ms, downstream electron acceptors, such as flavodiiron proteins, become active, leading to the reoxidation of P700 (Phase 2).

In Phase 1, the rate of P700 reduction decreased as the concentration of BQ increased. Increasing the BQ concentrations to 100 μM eliminated Phase 2 where the P700 could be reoxidized by the downstream electron sinks. These results suggest a slowdown in both the electron flow from PSII to PSI during Phase 1 and the electron flow from PSI to the downstream sinks. Further considering the enhanced ratio of PQ to PQH_2_ caused by the addition of BQ, it is likely that BQ is either interacting and extracting photosynthetic electrons from the electron carriers between the PQ pool and PSI, or competing with PQ for the photosynthetic electrons from PSII. The latter case is more likely, since it was also hypothesized in literature that BQ is preferably competing with PQ to obtain electrons from the Q_A_/Q_B_ sites of photosystem II because of the chemical structure similarity.[^2^, ^29^, ^33^]

The CoBP causes similar effects on the P700 as BQ, though at a lower amplitude (Figure 5d,e). As the concentration of CoBP increases, it results in a slower rate of P700 re‐reduction. The phenomenon could be caused by several different scenarios, as it was a result of the relative balance of the electron flow into the P700 and that out of the P700. However, the most likely reason is a strong decrease of the upstream electron flow (cytochrome b_6_f complex) to PSI. As shown in Figure 2, the growth of *Synechocystis* would be completely inhibited at a concentration of CoBP of >= 50 μM. Thus, it could be safely assumed that, under the PAM testing conditions, the metabolic electron sink demand after the addition of CoBP would be significantly decreased. Therefore, the most likely reason for the decreased rate of P700 re‐reduction is the decreased electron flow from Cyt 6bf into PSI; this remains to be further tested.

However, it seems that CoBP causes two distinct effects on the electron transfer chain between PQ and PSI. At low concentration (50 μM), CoBP oxidized the PQ pool similar as BQ, which suggests an electron sink effect of the chemical. At higher concentrations (e.g. 500 μM), CoBP, in contrast, led to a higher PQH_2_/PQ ratio. This suggests an inhibitory effect of CoBP on the electron flow from PQH_2_ to PSI, likely by affecting one or more components (i.e., cytochrome b_6_f complex, plastocyanin) of this pathway. The overall performance of CoBP is likely a balance between the sink and the inhibitory effects, which is dependent on the concentration.

In summary, BQ and CoBP affect the growth and electrogenic activity of *Synechocystis* cells via distinct mechanisms, although both of them give similar short‐term current output patterns and inhibit the growth at concentrations of above 50 μM. The toxicity of BQ is likely due to its interruption of the photosystem by interacting with electron carrier(s) between PSII and PSI. In contrast, CoBP appears to inhibit electron flow from the PQ pool to PSI, potentially causing an over‐reduction of the electron transport chain in the thylakoid membrane, especially at high concentrations. This disruption may lead to a shortage of electrons for downstream carbon fixation, further limiting cellular growth.

### 2.4 Direct Electron Extraction from the Photosynthetic Electron Transfer Chain

**Figure 6:**
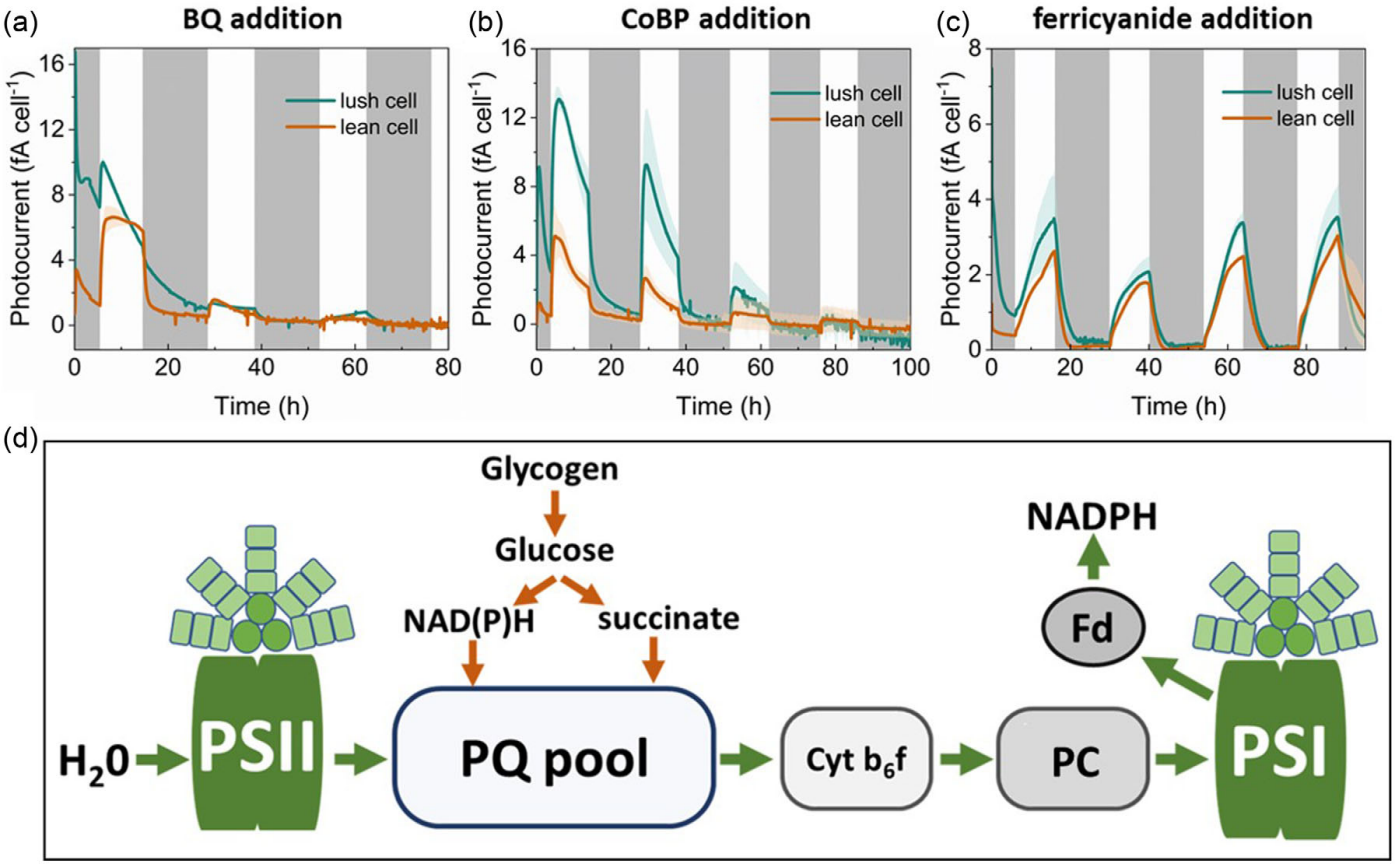
Current generation of BPV under circadian illumination (10 h light/14 h dark) and schematic diagram of the electron transfer chain. a) Current generation of lush and lean cells under a light/dark cycle with 20 μM BQ as the mediator. b,c) Current generation of lush and lean cells under the same cycle with 100 μM CoBP and 200 μM ferricyanide, respectively. The BPV systems are inoculated at 0 h. In the figures, the gray areas represent the dark phase, while the white areas indicate the illumination phase. Means and standard deviations are presented (*n* = 3). d) Schematic diagram of the photosynthetic and respiratory electron transfer chains. Green arrows indicate photosynthetic electron flow, and orange arrows represent respiratory electron flow. The growth profiles under these test conditions are given in Figure S3, Supporting Information, including the cell numbers that are used for calculating the single‐cell electrogenic activity.

The mediators were further tested in circadian cycles in the BPV reactors, as continuous illumination might cause energy overflow and oxidative/reductive stress to the cells. This could affect the performance of the mediators in the BPV system. Also, the light‐dark cycle is closer to the natural condition, where BPVs are expected to operate in the future. To exclude or evaluate the potential interference of the electron source coming from carbon turnovers, we prepared *Synechocystis* precultures under circadian illumination to minimize the intracellular storage carbon levels (the so‐called “lean cells” in this work) and compared them to the precultures cultivated under continuous illumination (i.e., “lush cells”) for the performance in BPV. Lean cells do not have an active contribution of the electrons from storage carbon, whereas lush cells can utilize the storage carbon and feed electrons into the photosynthetic electron transfer chain via PQ pool (**Figure** **6**d).

In general, the lush cells generate higher current output compared to the lean cells (Figure 6a–c), indicating the contribution of storage carbon to the photocurrent output,^[^ ^34^ ^]^ particularly for the case of CoBP. But the differences are reduced with consecutive circadian cycling. Dark currents also are stabilizing on lower levels approaching almost zero in the case of ferricyanide (Figure 6c), while the currents immediately rise sharply when light is turned back on. On the other hand, dark currents drop over hours after switching light off, maybe pointing to a contribution of metabolism of storage compounds built up again during the illumination phases. This demonstrates that the content of storage carbon (and the corresponding electron flux originating from this carbon turnover) is not essential for the EET process under circadian illumination, but it can contribute to the amplitude of current output and also provide some power output after lights are off.

Simultaneously, the growth of *Synechocystis* cells in these three systems was also recorded (Figure S5, Supporting Information). In the case of ferricyanide, cells were able to grow and maintain a stable current output.

The electrogenic activities of *Synechocystis* lasted for slightly longer under the light‐dark cycles compared to that under continuous illumination, while using BQ or CoBP as the mediator. Less illumination stress seemed to prolong the microbial metabolic activity, as well as the chemical stability in the case of BQ. More importantly, the lean cells also showed a light‐dependent current output pattern, despite a decreasing current density only detectable over a few cycles. These results show that both BQ and CoBP could extract electrons directly from the photosynthetic electron transfer chain, the same as suggested by the PAM and DUAL‐KLAS‐NIR measurements, and, more importantly, also suggest that these two mediators could potentially be promising options for BPV systems in the future, although their inhibitory and disruptive effects on cellular growth and photosynthetic electron transfer would need to be identified and eliminated.

## 3 Conclusion

Mediators play a critical role in promoting the electrogenic activity of most cyanobacterial strains. In this work, we systematically investigated and compared the performance of three mediators with different physicochemical properties in BPV systems, i.e., BQ, CoBP, and the commonly used mediator ferricyanide. The effects of the mediators on the growth and electrogenic activity of *Synechocystis* were systematically characterized, and PAM and DUAL‐KLAS‐NIR were used to assess their impacts on the redox status of the PQ pool and PSI. The results suggest that ferricyanide is so far the best option, considering the overall current output density and stability. However, BQ and CoBP have great potential to be promising alternatives in the future, as both of them promote higher current output than ferricyanide. Particularly, the current output with BQ is double that with ferricyanide. The remaining issue to be solved is the short‐term current output and strong inhibitory effects on cell growth caused by these two chemicals. Deep analysis of the photosystem indicates that the mechanism for BQ is primarily due to its strong oxidizing effect on the photosynthetic electron transport chain and its chemical instability under BPV conditions, while CoBP probably inhibits the electron transfer from the PQ pool to PSI. Further studies are required to reveal the molecular mechanism and to develop robust strains (or screen for alternative strains) that can resist the toxic effects of these chemicals better. On the other hand, a chemical synthesis approach could also be applied to create derivatives of these two chemicals, which might lower toxicity to the cells while maintaining their high capability to mediate the EET pathway for *Synechocystis*.^[^ ^4^, ^32^, ^35^ ^]^

## Conflict of Interest

The authors declare no conflict of interest.

## Supporting information

Supplementary Material

## Data Availability

The data that support the findings of this study are available from the corresponding author upon reasonable request.
