# Supplementary material for: The Impact of Redox Mediators on the Electrogenic and Physiological Properties of Synechocystis sp. PCC 6803 in a Biophotovoltaic System
Source: ChemSusChem. 2025 Apr 25;18(13):e202402543. doi: 10.1002/cssc.202402543 (PMC12232079; doi:10.1002/cssc.202402543)
Supplement: Supplementary file 1 — Supplementary Material [file CSSC-18-e202402543-s001.pdf]

Supporting Information

The impact of redox mediators on the electrogenic and physiological properties of *Synechocystis sp.* PCC 6803 in a biophotovoltaic system

Jianqi Yuan[a], Yu Bai[a], Claudius Lenz[a], Vincent Reilly-Schott[a], Hans Schneider[a], Bin Lai*[a], Jens Olaf Krömer[a]

[a] Department of Microbial Biotechnology
Helmholtz Centre for Environmental Research - UFZ
Permoserstraße 15, 04318 Leipzig, Germany
E-mail: bin.lai@ufz.de

Table of Contents

1 Materials and Methods 1
1.1 Strains and cultivation 1
1.2 BPV reactor construction and operation 1
1.3 Cell growth measurement 1
1.4 Mediator characterization 2
1.5 State transition status determination 2
1.6 DUAL-KLAS-NIR measurement 3
2 Supplementary Tables 4
3. Supplementary Figures 5
Figure S1 Cyclic voltammogram of CoBP and concentration change of ferricyanide 5
Figure S2 Emission spectra of white LED used for illuminating biophotovoltaic reactors 6
Figure S3 Schematic diagram of state transition. 7
Figure S4 Measurement of state transition status using Multi-Color PAM. 8
Figure S5 Growth of lush and lean *Synechocystis* under circadian illumination with three different mediators at specific concentrations. 9
Figure S6 Illustration of BPV reactor. 10
Figure S7 Comparation of BQ concentration variation over 4 consecutive days in BPV systems detected by two different detectors. 11
Figure S8 HPLC-UV chromatograms at 280 nm for biotic control BPV reactors with 1 mM ferricyanide as mediator instead of BQ. 12
Figure S9 UV absorption spectra of BQ in nBG11 medium under various conditions. 13
References 14

1 Materials and Methods

1.1 Strains and cultivation

Synechocystis sp. PCC 6803 (*Synechocystis*) was purchased from the Pasteur Culture Collection of Cyanobacteria (Paris, France). For reactivation, cryo-preserved cells were plated on BG11 agar plates [1] and incubated in a photo incubator (Percival Scientific, Inc., IA, USA) for about 1 week at 30 ℃, 75% relative humidity, 50 µmol photons·m^{-2}·s^{-1}. After that, colonies from plates were transferred into baffled shake-flasks containing 50 ml nBG11 medium, a modified BG11 optimized for BPV cultivation [2]. To obtain cells that contain high intracellular glycogen levels (lush cells), liquid cultures were kept in a photo incubator (MultitronPro, INFORS AG, Bottmingen, Switzerland) at 150 rpm with 25 mm orbital throw, 30 ℃, 75% relative humidity, ambient CO_2, and continuous white light at 50 µmol photons·m^{-2}·s^{-1}. To obtain cells with minimized glycogen levels (lean cells), the liquid cultures were subjected to a circadian illumination cycle (10 hours light, 14 hours dark) with the other cultivating conditions the same as those for lush cells. After incubation for one week, cells were harvested by centrifugation (6000 g, 5 min, 25°C) at an optical density (OD_{750}) of about 3.5. Lean cells were harvested at the end of a dark phase. The cell pellets were resuspended in fresh nBG11 medium and inoculated into BPV reactors with sterile syringes.

1.2 BPV reactor construction and operation

A well-defined BPV setup was used for BPV tests in this work (Figure S6). This has been described in detail elsewhere [2]. Briefly, a CTAB-pretreated carbon cloth (1071HCB, The Fuel Cell Store, USA) with 12.5 cm^2 projected surface area was used as a working electrode. Stainless steel mesh (FE6210, Advent Research Material, England) and Ag/AgCl/KCl_{sat} electrode (RE-1CP, Als, Japan; 0.197 V vs SHE) were applied as the counter and reference electrodes, respectively. Working and counter electrode chambers were separated by a proton exchange membrane (diameter 9 mm, CMI-7000, Membranes International INC., USA).

The working volume was set to 240 ml. The working chamber of the BPV reactors was filled with nBG11 medium and was inoculated to a starting optical density OD_{750} of approximately 0.35, unless stated otherwise. The catholyte also consisted of nBG11 medium. The reactors were illuminated with white light LED jackets wrapping around the working electrode chamber. The light intensity was controlled at 100 µmol photons·m^{-2}·s^{-1} by constant voltage supply to the LED jacket using an AC-DC power station. The light spectrum was determined by using a spectroradiometer (CSS-45-WT, Gigahertz-Optik GmbH, Germany). The temperature was maintained at 30 °C by a water-recirculating thermostat. Cell precipitation was prevented by magnetic stirring at 200 rpm. The entire reactors were placed into Faraday cages made of solid stainless-steel plates, firstly to prevent electromagnetic noise from disturbing the measurements and secondly to precisely control the light input into the reactor by preventing penetration of ambient light. Depending on the different experiments, the working chamber medium also contained different concentrations of mediators, as described below. A potentiostat (VMP3, Bio-Logic, USA) was used to control the working electrode potential at 0.697 V (versus SHE; same for all potential values below unless otherwise specified) and record the current response. The BPV reactors were sampled daily for off-line measurements.

1.3 Cell growth measurement

The optical cell density was measured at a wavelength of 750 nm (OD_{750}) using a spectrophotometer (Libra S11, Biochrom, Cambridge, UK). Since the cell morphology is highly dynamic for photoautotrophs among different growth phases, we also measured cell number and

averaged cell size simultaneously, using a Coulter Counter (Multisizer 3, Beckman Coulter, Brea, USA). In addition, chlorophyll a (Chl_a) concentration was also measured following the protocol described by Zavrel et al [3]. Briefly, the cell pellets were harvested by centrifugation (4 °C, 17,000 g, 10 min), and the Chl_a was extracted by adding methanol. After extraction at 4 °C for 20 min, the supernatant was collected by centrifugation as above, and the Chl_a and carotenoid concentrations were measured at three wavelengths of 470 nm, 665 nm, and 720 nm.

1.4 Mediator characterization

The concentration of ferricyanide was determined by measuring the supernatant at 420 nm, after removing cells by centrifugation at 17000 g for 6 min.

1,4-benzoquinone (BQ) and its reduced form hydroquinone (hereafter as BQH_2) were determined using a reversed-phase high-performance liquid chromatography (HPLC) system (Vanquish, Thermo Fisher Scientific, Massachusetts, USA) equipped with a UV detector and a C18 column (RSLC 120, 2.2 µm, 2.1 × 150 mm, Thermo Fisher Scientific). The detection wavelengths were set to 280 nm for BQH_2 and 210 nm for BQ, respectively. The column temperature was set to 35 °C. The injection volume was 5 µl. The mobile phase was composed of an aqueous 0.1% (v/v) trifluoroacetic acid solution (A) and acetonitrile (min. 99.97 %) (B). A linear gradient elution was performed using the following program: 0 min, 10% B; 4 min, 15% B; 7 min, 100% B; 10 min 100% B; 11 min, 10% B; 14.5 min 10% B. The flow rate was kept constant at 0.6 mL min^{-1}.

The results of the HPLC method for BQ quantification were confirmed using liquid chromatography–mass spectrometry (LC-MS) (Figure S7), and the biological background noise was excluded as well (Figure S8). Samples were analyzed directly on a Vanquish Flex / Exploris 240 LC-MS system (Thermo Fisher Scientific, Massachusetts, USA), with an injection volume of 1 µl. The same column and flow rate were used as in the HPLC method mentioned above. However, minor adjustments to the chromatographic conditions were required for compatibility with MS operation: The mobile phase was modified to aqueous 1% (v/v) formic acid solution (A) and acetonitrile (min. 99.97 %) (B). A linear gradient elution was performed using the following program: 0 min, 5% B; 2.5 min, 10% B; 3.5 min, 100% B; 7 min 100% B; 8 min, 5% B; 11.5 min 5% B. Detection was performed sequentially in a single run using full-spectrum UV-vis (210–600 nm) and MS. With these LC-MS conditions, UV-quantification at 210 nm was not possible due to putative detector saturation by formic acid, so BQ was evaluated at 246 nm instead. MS conditions were set as follows: ESI positive ion mode ionization at 320 °C and 3.5 kV, single-ion monitoring (SIM) was used for BQ detection (109.0284 ±0.5 m/z), with microscan averaging over10 scans. Appropriate extracted ion chromatograms of the SIM data were used for MS peak quantification.

The relative concentration change (i.e. the degradation or chemical stability) of $[Co(bpy)_3]^{2+}$ (CoBP) was estimated by using cyclic voltammetry (CV) between day 0 before inoculation and day 5 after the end of the batch. The CV potential range was set between -0.203 V to +1.197 V at a scan rate of 50 mV s^{-1}. Each CV was repeated for 10 cycles. The voltammograms obtained by CV at day 0 and day 5 were compared to evaluate the stability of the mediator in the BPV system.

1.5 State transition status determination

State transition of the photosystem was evaluated using Multi-Color Pulse Amplitude Modulation (PAM) fluorometry (Walz, Germany) following the protocols reported by Appel et al [4]. The method has been previously documented in detail [5]. Specifically, the built-in red LED served as both the measuring and actinic light, with the measuring light intensity and gain set to 1. The procedure

involved initially turning on the measuring light to determine F_0, followed by a saturating pulse at 3500 μmol photons·m⁻²·s⁻¹ applied to a 1.2 ml *Synechocystis* culture (cell density of 2.5 μg Chl_a ml⁻¹). Next, the sample was exposed to red actinic light at 110 μmol photons·m⁻²·s⁻¹ for 3 minutes, followed by another saturating pulse to determine Fm^-. During the subsequent 30 seconds of darkness, mediator was added to reach a certain desired final concentration. H_2O was added for the blank control. After an additional 3 minutes of red actinic light, a final saturating pulse was applied to measure Fm^+. The state transition was then calculated using Equation S1:

$$(F_{m^-} - F_{m^+})/F_0 \quad \text{(Eq.S1)}$$

1.6 DUAL-KLAS-NIR measurement

The absorption spectroscopy of P700 (photosystem I, PSI) was assessed using the DUAL-KLAS-NIR system (Walz, Germany). The detailed operational procedure has been described previously [5].The differential model plots for deconvolution were derived based on established methods [6]. Measurements were conducted in a cuvette containing 1.2 ml of *Synechocystis* at a final cell density of 20 μg Chl_a ml⁻¹, with different concentrations of mediators. For each sample, four wavelength pairs were equilibrated, and calibration was performed before recording data over 3 seconds. After the first second, a red-light pulse at 1350 μmol photons·m⁻²·s⁻¹ was applied for 600 milliseconds. The resulting plot represents the average of 16 measurements, each separated by 30 seconds of darkness.

2 Supplementary Tables

Table S1 Growth kinetics of *Synechocystis sp* PCC6803 in nBG11 medium with different mediators. The kinetics are corresponding to the data in Figure 2.

Mediator	Concentration (μM)	Maximum growth rate_OD750 (d^{-1})	Maximum growth rate_cell number (d^{-1})	Source
**BQ**	20	0.32 ± 0.04	0.38 ± 0.02	This work
	10	0.35 ± 0.03	0.45 ± 0.03	This work
**CoBP**	5	0.16 ± 0.00	0.26 ± 0.06	This work
**Ferricyanide**	500	0.19 ± 0.03	0.36 ± 0.02	This work
	200	0.26 ± 0.01	0.35 ± 0.04	This work
	100	0.25 ± 0.01	0.38 ± 0.01	This work
	20	0.28 ± 0.02	0.43 ± 0.02	This work
**No mediator**	-	1.29 ± 0.10	1.42 ± 0.06	(Lai, B. et al) [2]

3. Supplementary Figures

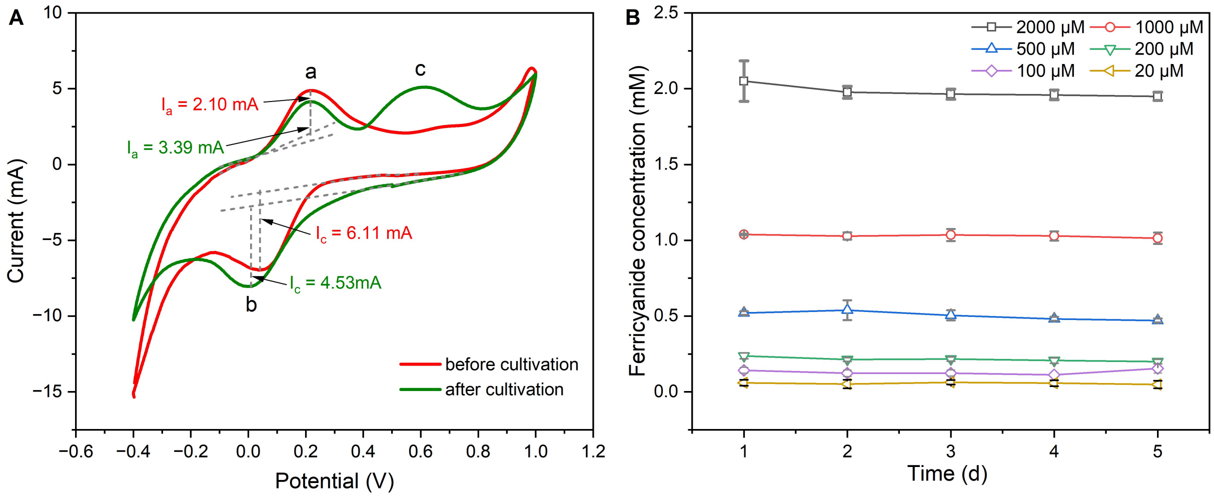

Figure S1 Cyclic voltammogram of CoBP and concentration change of ferricyanide. (a) CV curve of CoBP measured before and after 4 days of BPV operation. Peak a and b represent the oxidization and reduction peak of CoBP, respectively. Peak c is highly likely caused by the secreted metabolites of *Synechocystis* cells after 4 days' cultivation. (b) Variation in ferricyanide concentration during 4 days of BPV operation with different initial ferricyanide concentrations. Means and standard deviations are presented (n = 3). Before cultivation, the anodic (I_a) and cathodic (I_c) currents are 2.10 mA and 6.11 mA respectively; after cultivation, the anodic (I_a) and cathodic (I_c) currents are 3.39 mA and 4.53 mA respectively. The total current drops by 3.6%, from 8.21 mA for the case of before inoculation to 7.92 mA for the case of after cultivation. The data was processed using Origin 2024b.

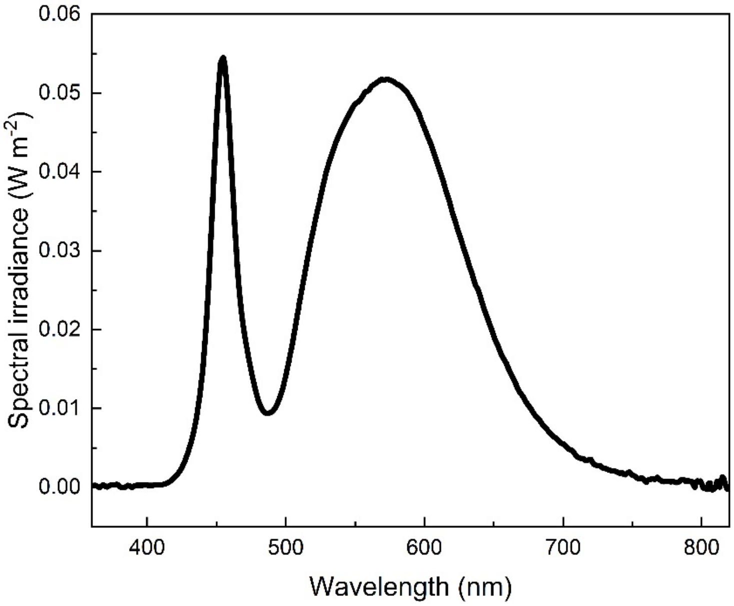

Figure S2 Emission spectra of white LED used for illuminating biophotovoltaic reactors.

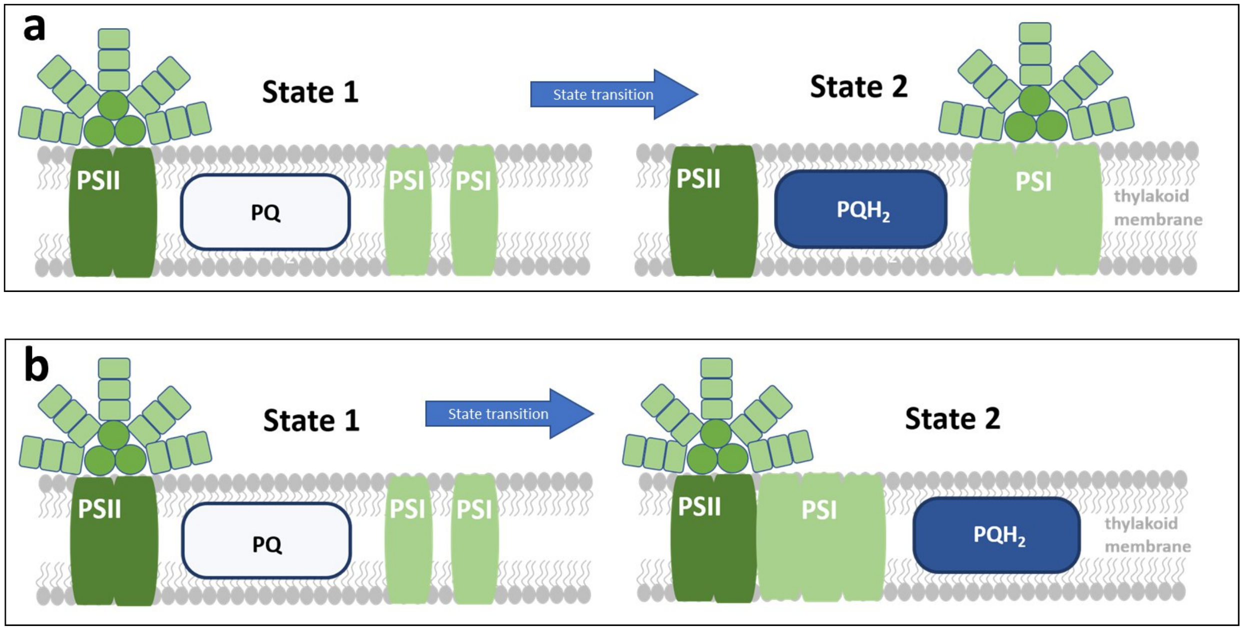

Figure S3 Schematic diagram of state transition. (a) The phycobilisome movement model: In State 1, the phycobilisome is attached to PSII. When PQ pool becomes reduced, it shifts to State 2, where the phycobilisome moves to PSI. (b) The spillover model: Upon reduction of the PQ pool, the PSII-phycobilisome complex moves closer to PSI, facilitating energy spillover from PSII to PSI through their interaction.

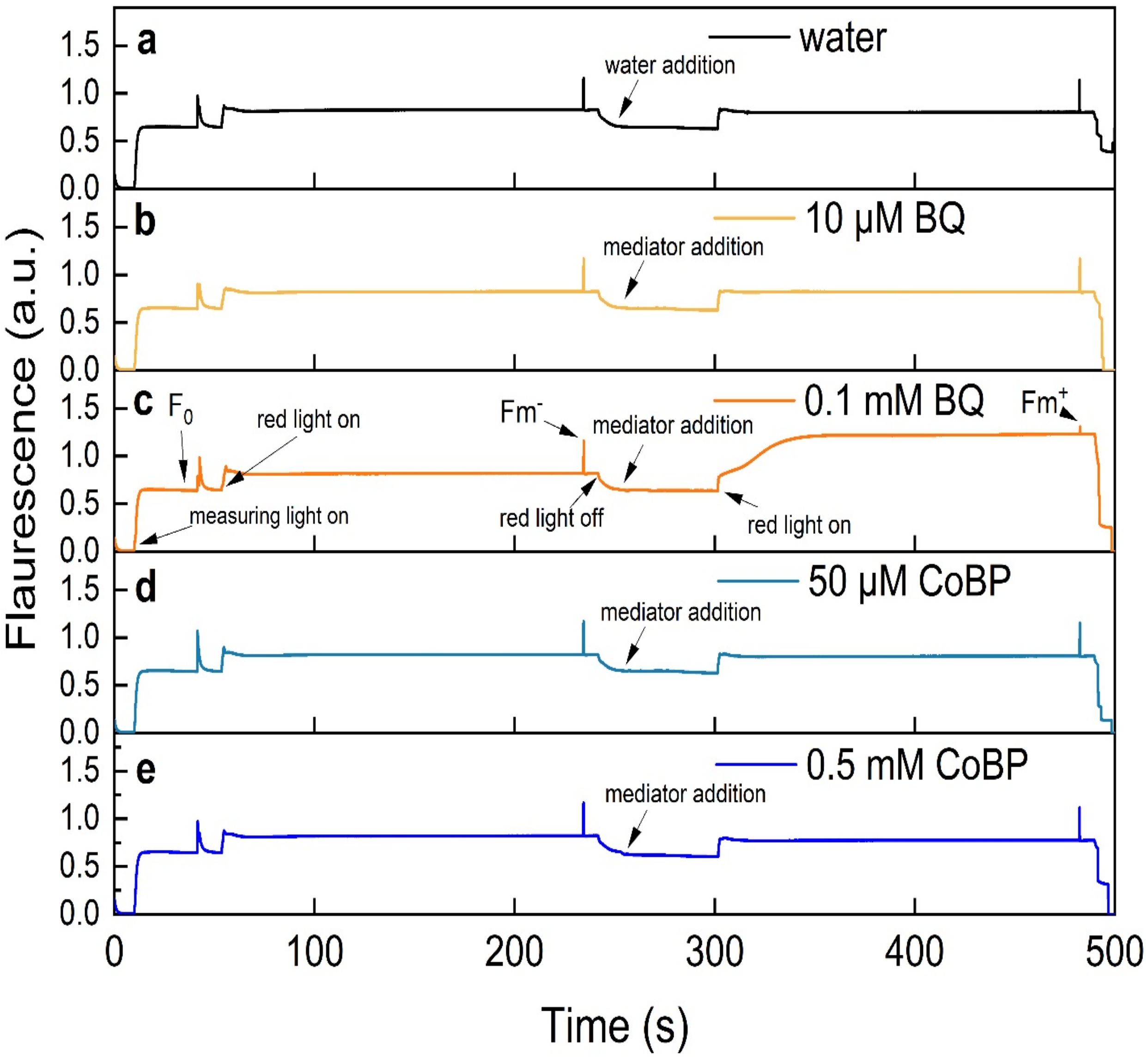

Figure S4 Measurement of state transition status using Multi-Color PAM. During the 30-second dark period, different mediators were added to achieve the desired final concentrations. (a) H_2O was added as a blank control. (b)–(e) The same volume of mediators was added to reach final concentrations of 10 μM BQ, 0.1 mM BQ, 50 μM CoBP, and 0.5 mM CoBP, respectively. The detailed procedure is illustrated in panel (c). All traces represent the average of five replicates.

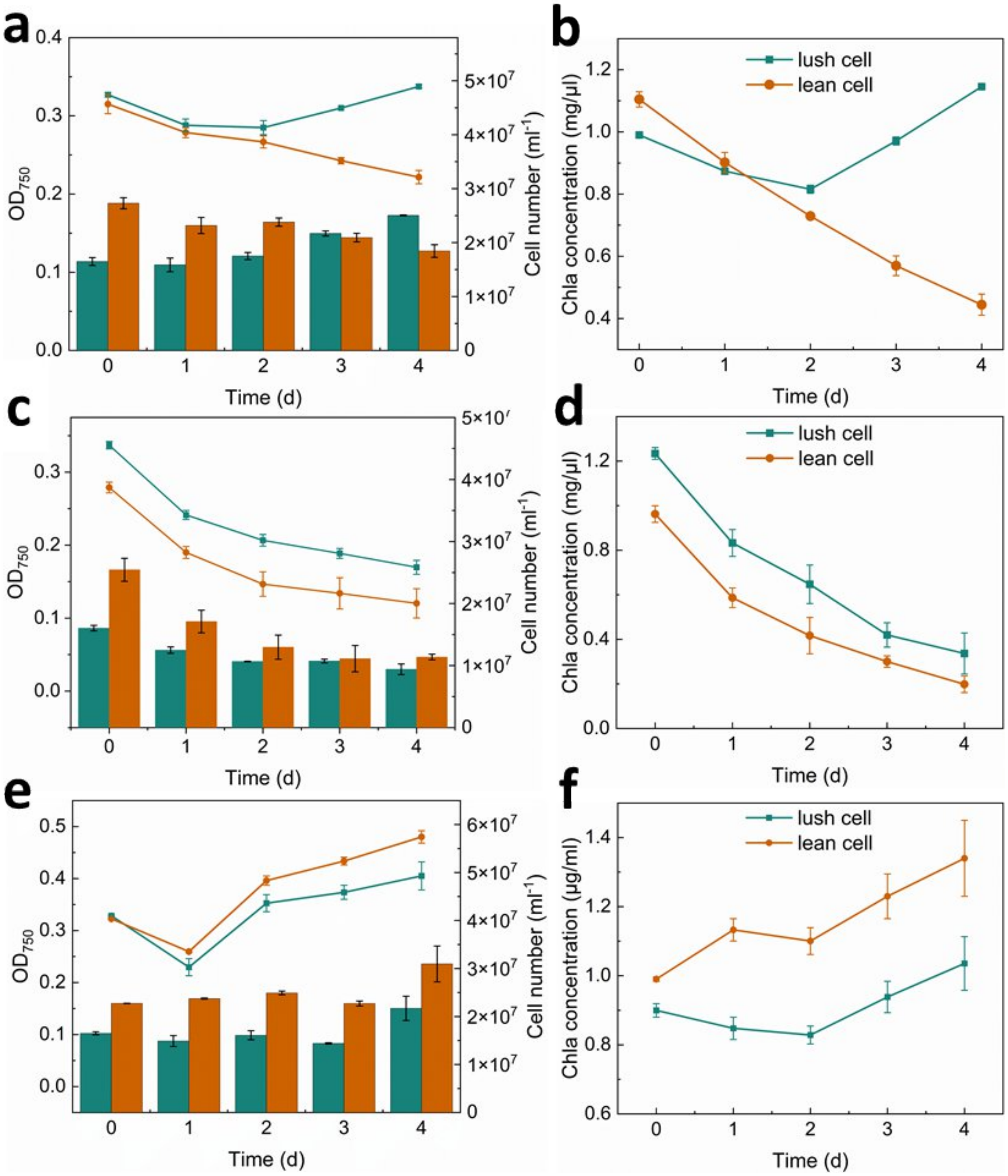

Figure S5 Growth of lush and lean *Synechocystis* under circadian illumination with three different mediators at specific concentrations. Illumination followed a 10-hour light/14-hour dark cycle. Experiments were run for four days. Data for lean cells is provided in orange and data for lush cells is shown in green. (a) OD_{750} (line and symbol) and cell number (bar) with 20 μM BQ. (b) Chl_a concentration (line and symbol) with 20 μM BQ. (c) and (d) Changes in OD_{750}, cell number, and Chl_a concentration with 100 μM CoBP. (e) and (f) Changes in OD_{750}, cell number, and Chl_a concentration with 200 μM ferricyanide. Means and standard deviations are presented (n = 3).

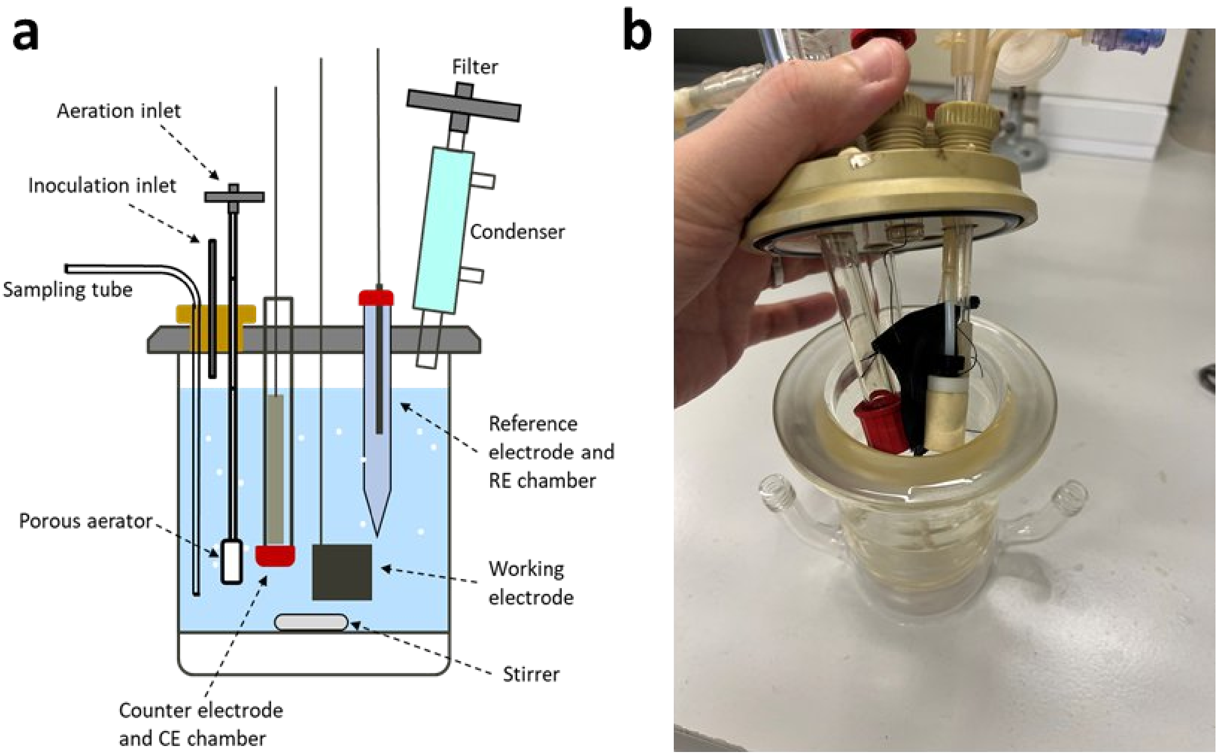

Figure S6 Illustration of BPV reactor. (a) Schematic drawing of BPV system. The reference electrode (RE) chamber was filled with saturated KCl solution inside and communicated with the bulk liquid through a porous glass frit inlaid at the bottom of the chamber. The counter electrode (CE) chamber which is open to the air is filled with sterile BG11 medium, and separated from the working chamber by a proton exchange membrane at the bottom. The sampling tube always connects to a sterile sampling bottle, and the inoculation inlet is sealed up by a cap unless inoculation. Additionally, the whole reactor always stands on a magnetic stirrer controlling the stirrer bar inside the reactor, and is shielded by Faraday box consistently. (b) Photograph of open and disintegrated reactor.

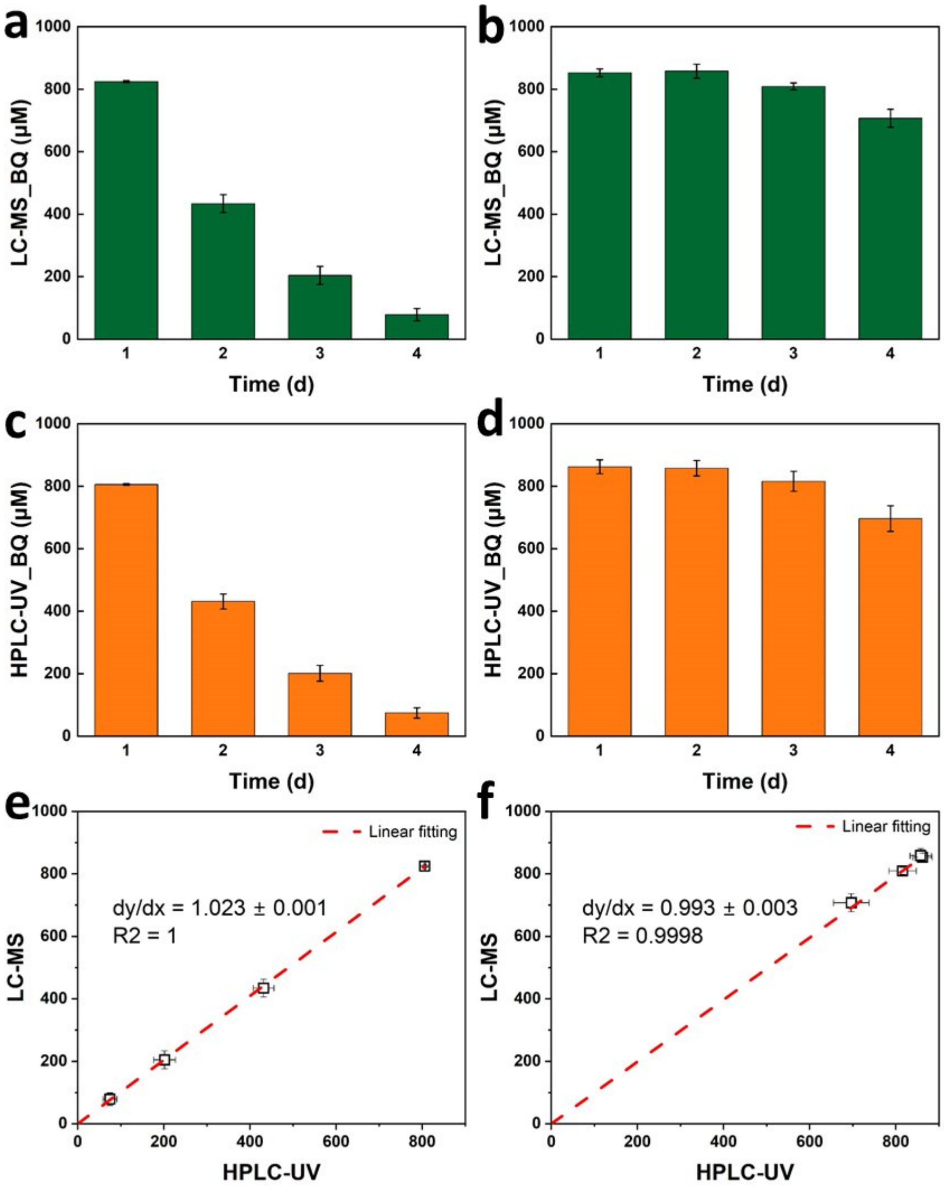

Figure S7 Comparation of BQ concentration variation over 4 consecutive days in BPV systems detected by two different detectors. (a) BQ concentration detected by mass spectrometry in BPV systems without potential bias under darkness; (b) BQ concentration detected by mass spectrometry in BPV systems with a 0.697 V potential bias under illumination; (c) BQ concentration detected by UV detector in BPV systems without potential bias under darkness; (d) BQ concentration detected by UV detector in BPV systems with a 0.697 V potential bias under illumination. (e), (f) Comparison of BQ concentrations detected by mass spectrometry and UV detection in BPV systems under the two respective conditions described above: (e) without potential bias in darkness, and (f) with a 0.697 V potential bias under illumination. Means and standard deviations are presented (n = 3).

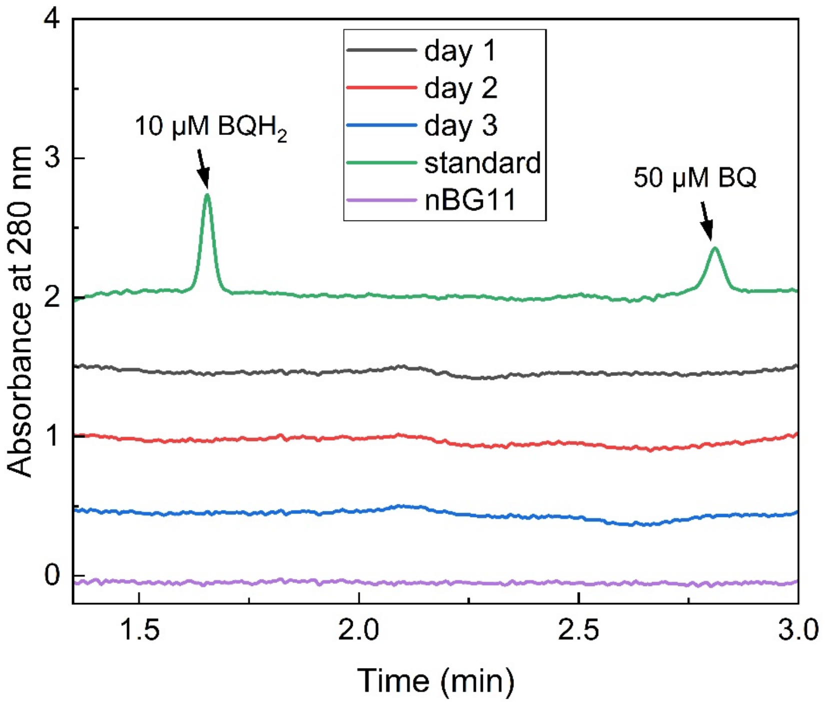

Figure S8 HPLC-UV chromatograms at 280 nm for biotic control BPV reactors with 1 mM ferricyanide as mediator instead of BQ. The green line is from nBG11 dissolved 10 μM BQH_2 and 50 μM BQ, which serves as standard sample. The violet line is from fresh nBG11 as blank control. The black, red and blue lines show the samples from 1 mM ferricyanide-mediated *Synechocystis*-driving BPV reactors taken from 3 consecutive days, respectively. Traces show the average of 3 replicates.

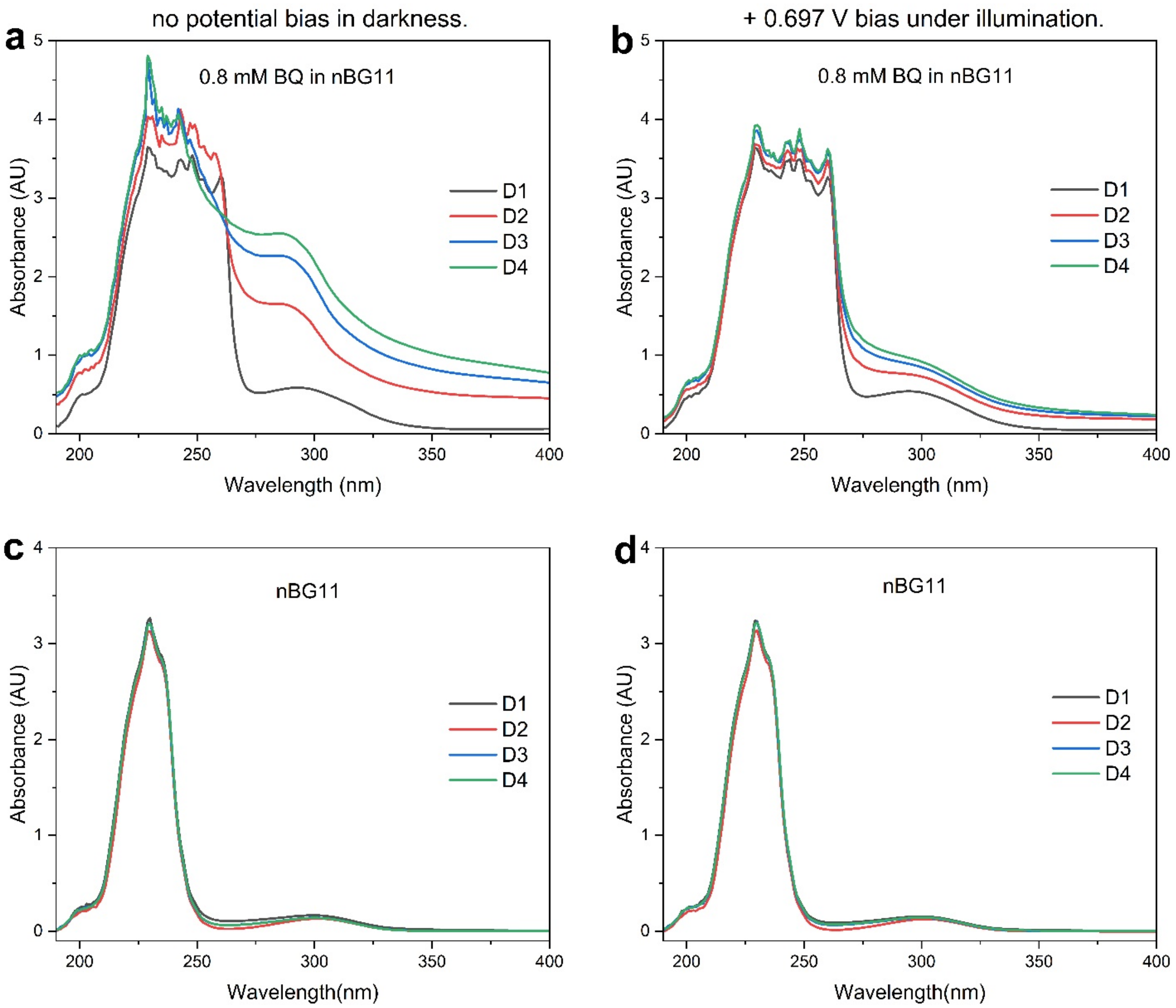

Figure S9 UV absorption spectra of BQ in nBG11 medium under various conditions. The solutions (0.8 mM BQ in nBG11 medium, or fresh nBG11) were incubated for 4 consecutive days in the BPV system with the following settings: (a) Incubation in darkness without applied potential. (b) Incubation illuminated at 100 μmol photons·m^{-2}·s^{-1} with 0.697 V bias. (c) nBG11 medium without BQ incubated in darkness without applied potential. (d) nBG11 medium without BQ illuminated at 100 μmol photons·m^{-2}·s^{-1} with 0.697 V bias.

References

[1] R. Stanier, R. Kunisawa, M. Mandel, G. Cohen-Bazire, *Bacteriol. Rev.* **1971**, *35*, 171-205.

[2] B. Lai, H. Schneider, J. Tschörtner, A. Schmid, J. O. Krömer, *Biotechnol Bioeng* **2021**, *118*, 2637-2648.

[3] T. Zavrel, M. Sinetova, J. Cerven, *Bio-Protocol* **2015**, *5*.

[4] J. Appel, S. Craig, M. Theune, V. Hüren, S. Künzel, B. Forberich, S. Bryan, K. Gutekunst, *Microorganisms* **2022**, *10*, 1617.

[5] J. Yuan, J. Appel, K. Gutekunst, B. Lai, J. O. Krömer, *Environ. Sci. Ecotechnol.* **2025**, *23*, 100519.

[6] a) M. L. Theune, S. Hildebrandt, A. Steffen-Heins, W. Bilger, K. Gutekunst, J. Appel, *Biochim. Biophys. Acta, Bioenerg.* **2021**, *1862*, 148353; b) C. Klughammer, U. Schreiber, *Photosynth Res* **2016**, *128*, 195-214.
